# Capacitance Variation Induced by Microfluidic Two-Phase Flow across Insulated Interdigital Electrodes in Lab-On-Chip Devices

**DOI:** 10.3390/s150202694

**Published:** 2015-01-26

**Authors:** Tao Dong, Cátia Barbosa

**Affiliations:** 1 Institute of Applied Micro-Nano Science and Technology, Chongqing Technology and Business University, Chongqing 400067, China; 2 Chongqing Engineering Laboratory for Detection, Control and Integrated System, Chongqing Technology and Business University, Chongqing 400067, China; 3 Department of Micro and Nano Systems Technology (IMST), Faculty of Technology and Maritime Sciences (TekMar), Buskerud and Vestfold University College (HBV), Borre 3184, Norway

**Keywords:** lab-on-a-chip, microfluidics, droplets capacitive sensing, interdigital electrodes

## Abstract

Microfluidic two-phase flow detection has attracted plenty of interest in various areas of biology, medicine and chemistry. This work presents a capacitive sensor using insulated interdigital electrodes (IDEs) to detect the presence of droplets in a microchannel. This droplet sensor is composed of a glass substrate, patterned gold electrodes and an insulation layer. A polydimethylsiloxane (PDMS) cover bonded to the multilayered structure forms a microchannel. Capacitance variation induced by the droplet passage was thoroughly investigated with both simulation and experimental work. Olive oil and deionized water were employed as the working fluids in the experiments to demonstrate the droplet sensor. The results show a good sensitivity of the droplet with the appropriate measurement connection. This capacitive droplet sensor is promising to be integrated into a lab-on-chip device for *in situ* monitoring/counting of droplets or bubbles.

## 1. Introduction

Microfluidic two-phase flow, especially droplet microfluidics, dealing with discrete droplets with a tiny volume at nanolitre and picolitre scale in lab-on-chip devices, has been widely used in the chemical, biological and medical areas, e.g., drug discovery [1], immunoassay [2], synthetic biology [3], cell analysis [4], cell culture [5], diagnostic testing [6], sample preparation [7,8], *etc*. Besides, differing from droplet microfluidics in which the droplets are generally manipulated in enclosed microchannels, digital microfluidics (DMF), despite of some disagreements about nomenclature, has been an emerging liquid-handling technology that enables individual control of droplets on an array of electrodes, which contrasts with the continuous nature of other microfluidic systems [9,10]. A typical example of DMF-based lab-on-chip devices is the continuous-flow PCR system [11], in which DNA samples are encapsulated with PCR reagents into tiny droplets, and flow through different temperature zones to implement programmed PCR cycles. Both droplet microfluidics and DMF take full advantage of tiny volumes with high area/volume ratio, which means more efficiency, less cost of reagents and lower energy consumption.

As one of the frequently used techniques for microfluidics detection, optical detection has been developed specifically for fluorescence, chemiluminescence, diffraction, absorption and refractive index variation [12–16]. Optical sensing systems, both “off-chip approach” and “on-chip approach”, have been used for detection and actuation of discreet droplets [12]. The off-chip approach requires macro-scale optical infrastructures such as high-speed charge-coupled device (CCD) cameras and microscopes, which have low background signal levels, however the sensitivity decreases due to the reduction in path length [13]. However, the involvement of macro-scale optical infrastructures results in more cost and less flexibility. The on-chip approach, known by the term micro-optical electromechanical systems (MOEMS), demands more integration of both fluidic elements and optical elements such as movable mirror arrays, refractive microlenses, and optical filters, *etc.* [15]. Consequently, it brings new system integration and process compatibility challenges for fabrication.

Alternatively, capacitive sensing offers another non-invasive solution to microfluidic detection. Considering a parallel-plate capacitor, the capacitance can be estimated by *C* = ε_0_ε_r_*A*/*d*, where ε_0_ is the vacuum permittivity; ε_r_ is the relative static permittivity of the dielectret; *A*, the overlap area and *d*, the distance between the parallel plates. Therefore, any change from the variables ε_r_, *A* and *d* can lead to a capacitance variation [17,18]. Chen *et al.* [19] were the first to investigate a miniaturized coplanar capacitive sensor for thermocapillary actuation of liquid films and droplets. Demori *et al.* [20] proposed a capacitive sensing system for two-phase flow of oil and conductive water in pipes. Ernst *et al.* [21] conducted the detection of microdroplets in flight by capacitive sensor, which allows for non-contact monitoring of a complete droplet dispensing process. Niu *et al.* [22] demonstrated a capacitive detection and sensing of droplets. Elbuken *et al.* [23] reported a commercially available capacitive sensor for detection of the presence, size and speed of microdroplet in microfluidic devices.

According to the review of the available literature, the currently available capacitive droplet sensing technique often operates based on the permittivity difference of fluids. Moreover, the capacitive sensor with coplanar configuration is compatible to the microfabrication processes, which make it possible to integrate the droplet sensor into the lab-on-chip devices, and thus to realize an on-line monitoring. However, it would be a challenge to have a capacitive droplet sensor for two liquids that have less difference in permittivity. Fortunately, such cases are very few; we did not find any droplets involving with two immiscible fluids that have the almost same permittivity.

In this work, a microfabricated capacitive sensor employing interdigital electrodes (IDEs) insulated by a thin film to detect water-in-oil or oil-in-water droplets in microchannels is presented. Both simulation work and experimental instigation on the capacitance variation have been conducted. Details of capacitive sensor design are described addressing the effective sensing area (active finger pairs) of the sensor. With diverse effective sensing length, the proposed capacitive droplet sensor can achieve good sensitivity. This sensor is promising for integration in a lab-on-chip device for droplet/bubble detection, as long as the two immiscible fluids have distinct permittivity.

## 2. Sensor design and Fabrication

### 2.1. Operational Principle

**Figure 1.**
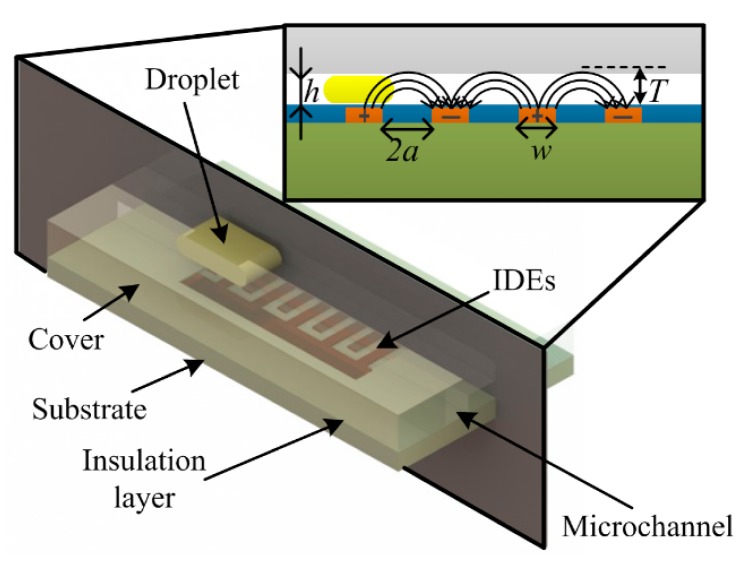
Structure of the sensing elements and cross-section of the sensor, showing how distinct materials cross the sensing area and the electrical field lines.

Microfluidic droplets are characterized by presence of two immiscible fluids: one is continuous while the other is discrete; for instances, oil droplets in continuous water and water droplets in continuous oil. If the involved fluids (phases) have distinct relative permittivity differences, droplet detection becomes possible by use of capacitive sensing. The capacitive droplet detector comprises of glass substrate, IDEs, insulation layer and microchannel cover, as schematically illustrated in Figure 1.

Like the parallel plate capacitor, the capacitance of the interdigital sensor depends on the properties of the material crossing the electrodes area and the geometrical parameters of the electrodes [24]. Chen *et al.* [19] deduced an equation to estimate the capacitance of an electrode pair:

(1) $$C=\frac{2\varepsilon_{r}\varepsilon_{0}l}{\pi }\ln\;\left[\left(1+\frac{w}{a}\right)+\sqrt{{\left(1+\frac{w}{2}\right)}^{2}-1}\right]$$

where *a* is the half width of the electrode gap; *w* is the width of electrode finger; and *l* is the overlap length of electrode pair. Equation (1) is valid for a finite value of *w*, with conditions *w*/*a* ≫ 1 and *l* ≫ *w*. It should be noticed that Equation (1) assumes a direct contact between fluids and electrodes without consideration of the substrate and the insulation layer. An analytical model for the IDEs capacitance with consideration of the multilayered structures has been developed by Igreja and Dias [25].

Differing from that of the parallel plate capacitor, the coplanar feature of IDE capacitor is more suitable for microfabrication processes. Additionally, the IDE capacitor increases the sensing area while keeping the area of electrodes unchanged compared to the parallel plate capacitor.

According to Figure 1, the electrical field lines generated by the electrode pair have a maximum penetration depth *T*, the maximum height measured perpendicularly to the electrodes plane. The penetration depth of an electrode pair can be determined by [19]:

(2) $$T=a\sqrt{{\left(1+\frac{w}{a}\right)}^{2}-1}$$

Consider a microchannel with a channel height *h*, which is filled with fluids. If *T* < *h*, the capacitance will not be affected by the top wall of the microchannel, however, when *T* > *h*, the influence from the top wall of microchannel upon the total capacitance has to be considered.

A thin insulation layer was introduced in the sensor to avoid a short circuit between the electrodes. The thickness of the insulation layer is a critical parameter for the sensor design. A thicker insulation layer will increase the initial capacitance of the sensor (while no droplet passes by) and reduce the capacitance variation (while droplet passing) thus decreasing the sensitivity. Similar consideration was also confirmed by Elbuken *et al.* [23]. From this point, a thinner insulation layer is preferred. However, a very thin insulation layer may cause bad insulation. Therefore, an appropriate thickness of the insulation layer has to be decided in terms of such compromise.

### 2.2. Structure and Materials

The capacitive droplet detector is composed of a basic three-layer structure with embedded IDEs. The 0.1 μm thick gold IDEs were patterned on a 200 μm glass substrate. A 2-μm SU-8 film coated the IDEs as an insulation layer to avoid direct contact between the fluids and IDEs. This thickness of SU-8 can provide reliable insulation meanwhile define a moderate reference capacitance, as discussed in [26]. At last, a 400 μm PDMS cover with a groove was bonded on the insulation layer to form a microchannel. The microchannel cross-section is 150 μm high and 600 μm width. The fluid inlets and waste outlet were mounted onto the PDMS cover during the casting process. The geometrical parameters of the IDEs are listed in Table 1.

**Figure 2.**
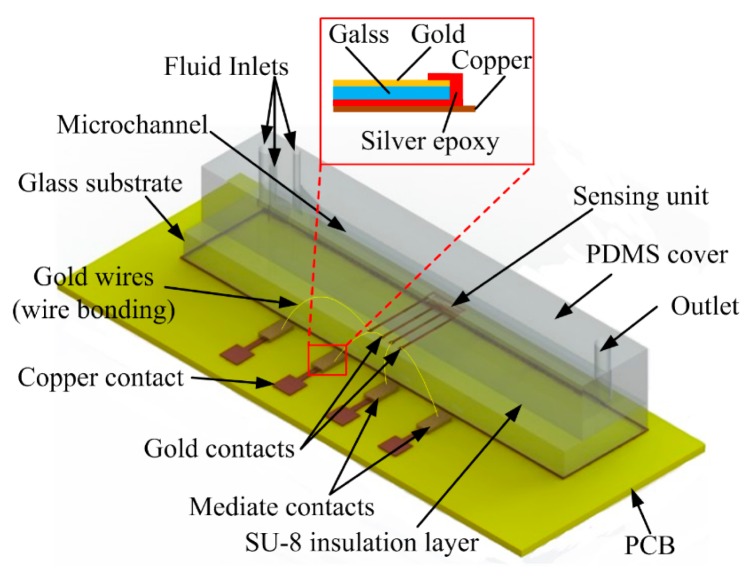
Schematics of the droplet sensor and connection to a PCB with mediate contacts (thin gold film on glass substrate). The inserted figure illustrates the electrical connection between the mediate contact and the copper contact on PCB through conductive silver epoxy.

To evaluate the influence of the number of finger pairs upon capacitance and further upon voltage values, we devised a droplet detector with different numbers of finger pair in the capacitive unit, which can be tested with different connections. The capacitive unit had four gold contacts allowing for outward connection. One of the gold contacts was electrically connected continuously, while the other three can be connected in proper ways to involve *n* (one connection), *2n* (two connections) or *3n* (three connections) active finger pairs (Figure 6a in section 4.2 shows more details of the measurement connection). Figure 2 illustrates the structure of the sensing unit and connection with the external circuit.

For convenience of signal acquisition, we used mediate contacts, which comprise thin gold films sputtered on a glass substrate. The mediate contacts were glued and electrically connected with the copper contacts by conductive silver epoxy, shown as inserted in Figure 2. Then we used wire bonding to connect the sensing unit to the mediate contacts.

### 2.3. Microfabrication

**Figure 3.**
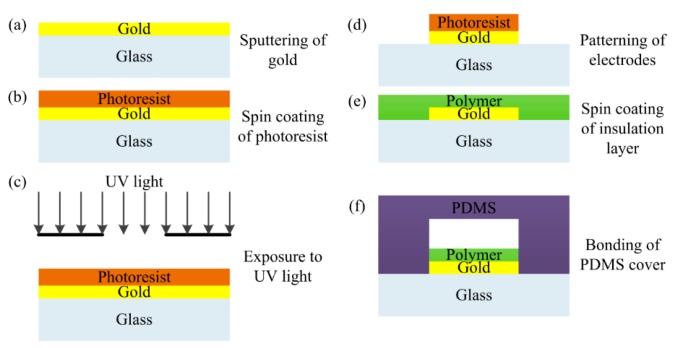
Microfabrication process flow: (**a**) Sputtering of gold on glass substrate; (**b**) Spin coating of photoresist on top of the gold film; (**c**) Exposure to UV light; (**d**) Patterning of gold electrodes; (**e**) Spin coating of insulation layer; (**f**) Bonding of PDMS cap to the processed wafer.

Sensor fabrication follows the steps presented in [26]. Firstly, the gold is sputtered on top of a glass substrate to form the electrodes layer (Figure 3a). Then a layer of negative photoresist is deposited using spin coating (Figure 3b). Subsequently, the wafer is exposed to UV light to remove photoresist with a mask containing the pattern of IDEs (Figure 3c).

With a lift-off process, gold IDEs are patterned (Figure 3d); after removal of the remaining photoresist, SU-8 polymer is spin coated on top of the electrodes to form the insulation layer (Figure 3e), whose thickness is well controlled as designed. The final step is to bond the PDMS cover to the processed wafer to form microchannels (Figure 3f). The microfabrication processes employed are very common. For this reason, the capacitive droplet sensor can be easily integrated into the existing lab-on-chip devices while the associated cost is kept low.

## 3. Experimental Setup and Test Procedure

**Figure 4.**
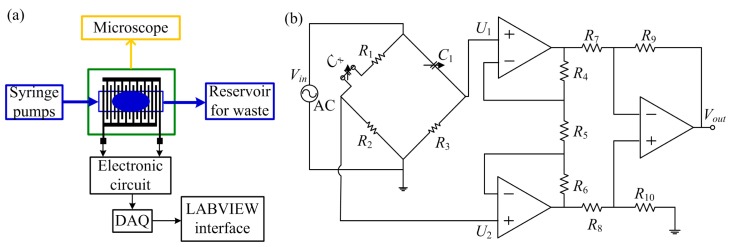
Schematic of the experimental setup. (**a**) Fluids are driven into the microchannel of sensor with two syringe pumps. The droplet flow pattern is visualized with a microscope. And the induced capacitance variation is transformed into voltage variation. Signal is acquired with a DAQ and finally displayed in a computer interface with LABVIEW; (**b**) Electric circuit for signal acquisition.

During experiments, we used a commercial domestic olive oil and deionized water, due to the fact that they have very distinct relative permittivities (80 for deionized water and 2.4 for olive oil), respectively. Two syringe pumps were used to drive the fluids into the microchannel; meanwhile, the precise flow-rate of fluids can be set in the syringe pumps. Tygon^®^ microbore tubing (Cole-Parmer, Vernon Hills, IL, USA) with outer diameter of 1.5 mm and inner diameter of 1.0 mm was used to connect the fluids from the syringe pump to the fluid inlets. The flow-rates of water and oil were well controlled to generate stable droplet flow. Before testing, the microchannel and connection tubes were flushed with liquids at high flow-rates for several minutes (e.g., 5 min), so that most of the air can be removed. Then the flow-rates of the liquids were adjusted to perform the regular tests. With this method, we can make sure there were no air bubbles inside the sensing area during the droplet detection test. For the first stage, tests were performed with water as continuous phase and oil as dispersed phase. Initially, the microchannel was filled only with water and afterwards the olive oil was injected at a constant flow rate. For the second stage, we used the same procedure with oil as continuous phase and water as dispersed phase, except that initially the microchannel was filled with olive oil. Inflow of the dispersed phase was via the center inlet while the continuous phase was via the lateral two inlets. To validate the experimental results, the droplet flow pattern was monitored through a Discovery v12^®^ microscope (Carl Zeiss Microscopy GmbH, Jena, Germany). To evaluate the influence of the number of finger pairs upon the capacitance of the sensor, the capacitive sensor was tested with one, two and three connection(s), respectively. The schematic on the experimental setup is presented in Figure 4a.

Due to the presence of droplets in the microchannel, a capacitance variation was induced, which was then transformed into voltage variation by use of a simple Wien bridge [27]. The voltage variation was amplified with an instrumentation amplifier, where the differential input potential were transformed into a single terminal output voltage [28]. During measurements, the Wien Bridge was driven by an AC voltage. Before starting test, the balance of the bridge was achieved by adjusting the variable capacitor *C*_1_ (Figure 4b). When the bridge was balanced, the differential inputs of the instrumentation amplifier, *U*_1_ and *U*_2_, shall be equal. Therefore, the output voltage will be zero, as indicated by [29]:

(3) $$V_{\text{out}}=\left(U_{2}-U_{1}\right)\left(1+2\frac{R_{4}}{R_{5}}\right)\left(\frac{R_{9}}{R_{7}}\right)$$

According to the electrical circuit in Figure 4b, the output voltage can be estimated as below, where *G* is the gain provided by the instrumentation amplifier:

(4) $$V_{\text{out}}=G(U_{2}-U_{1})=GV_{in}(s)\left(\frac{sR_{2}C_{x}}{1+s\left(R_{1}+R_{2}\right)C_{x}}-\frac{sR_{3}C_{1}}{1+sR_{3}C_{1}}\right)$$

Due to capacitance variation in the sensor, the values of the differential inputs will vary and the output will also change. The signal from the electronic circuit was acquired with a NI USB-6211 16-bit DAQ from National Instruments (Austin, TX, USA). A 5-volt AC signal to feed the Wien bridge was provided by the DAQ with a frequency of 1.0 kHz; Two operational amplifiers (OPA2107AU) worked together to realized the instrumentation amplifier circuit. After acquisition by an Express VI at sampling rate of 10 kHz rate, the signal was demodulated and filtered to reduce the noise interference. Finally, the signal was displayed in a LABVIEW interface.

## 4. Results and Discussion

### 4.1. Initial Capacitance of the Sensor—Simulation

To evaluate the initial capacitance of the droplet detector, we modeled the sensor configuration using the commercial software COMSOL Multiphysics^®^ version 4.3b (COMSOL AB, Stockholm, Sweden). During simulations, the AC/DC module and electrostatics interface were used. The initial capacitance was determined in terms of charge conservation, with one electrode set at 0 V and the other three electrodes set at 1 V. The different active connections (one, two or three) was modelled with different number of finger pairs (namely, 10, 20 and 30).

A 3D model including all the multilayer structures was built for the droplet sensor. Particularly, the IDEs were modelled as a 2D structure instead of 3D one. As the thickness of IDEs (0.1 μm) is much smaller compared to their width (50 μm) and length (850 μm), there is a large aspect ratio, resulting in troubles of meshing and dramatically increasing of computational load. The 2D simplification addressed the very thin film essence of IDEs and solved the meshing problem successfully, without affecting the simulation results. For the other multilayer structures, the real dimensions for each layer had been used for simulation.

The olive oil and deionized water were used as the different medium filled in the microchannel, to calculate the initial capacitance for different cases. Table 2 summarizes the initial capacitance measured under different liquid (continuous phase) and different number of active finger pairs.

According to Table 2, the initial capacitance shows an approximately linear dependence on the number of active finger pairs. Increasing the number of active finger pairs results in a larger initial capacitance, which means the density of electrical field lines on top of the sensing area is increased. Besides, the material of the continuous phase in the microchannel also influences the initial capacitance. The initial capacitance with deionized water is much larger than that with olive oil, due to the fact deionized water has a considerably larger relative permittivity compared with olive oil.

The initial capacitance is of great interest for the operation of the sensor. It should be detectable by the acquisition circuit, while on the other hand, it should not be so large that a small capacitance variation caused by the droplet can't be detected by the same circuit.

**Figure 5.**
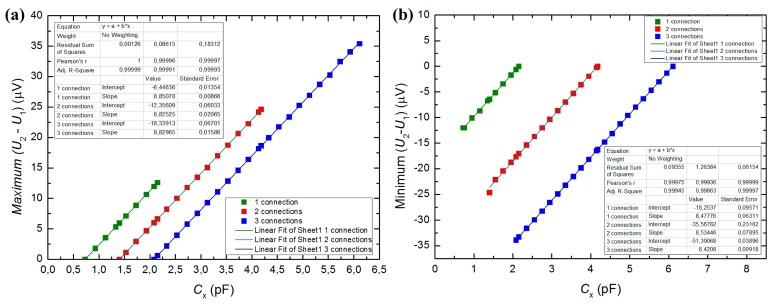
(**a**) Expected maximum output voltage variation, with oil as continuous phase; (**b**) Expected minimum output voltage variation, with water as continuous phase.

When the Wien bridge in the acquisition circuit is balanced, the output voltage should be zero, as the initial capacitance was balanced by *C*_1_. When the droplet passes by, the output voltage varied with respect to the variation in *C_x_*. According to the initial capacitances listed Table 2, the output voltage was simulated for different values of *C_x_* by NI Multisim, as plotted in Figure 5. The eventual losses due to stray capacitances or bad circuit shielding were not considered. Additionally, the linear fitting of the data, including the slope and intercept parameters values is presented in Figure 5.

Figure 5 clearly shows the range of output voltage with respect to the capacitance variation that could be induced by droplets with different dimensions, based on the initial capacitance and active finger pairs. According to Figure 5, there is a linear response of the output voltage with the capacitance C*_x_*. With these linear relations, it is possible to determine the capacitance variation induced by droplets with different sizes. Read the output voltage from the circuit and apply the value to the plots in Figure 5, we can estimate the induced capacitance value.

It should be noticed that the plots in Figure 5 did not account the existence of stray capacitances, which make it more difficult to balance the Wien bridge in practical tests.

### 4.2. Capacitance Variation Simulation—Oil-in-Water Droplets

**Figure 6.**
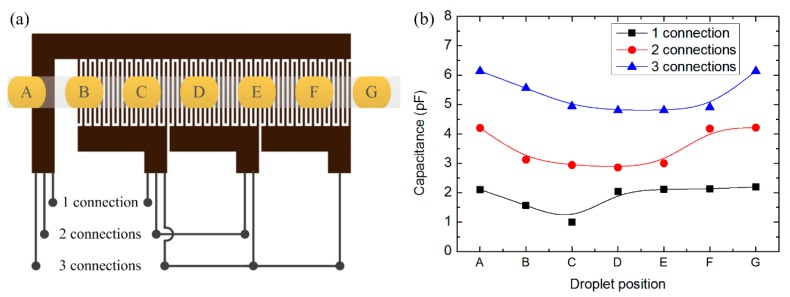
Capacitance variation induced by a 1.6 mm long oil droplets in water: (**a**) droplet positions (**A**–**G**) relative to the sensor; the active number of finger pairs was changed with 1, 2, and 3 connections; (**b**) capacitances in response to different droplet positions.

To reveal the direct dependence of capacitance variation upon the presence of droplet, simulation work on the sensor with diverse positions of oil droplet in water was performed. The typical results are shown in Figure 6, in which the influence from the number of active finger pairs is also included.

According to Figure 6, the capacitance varies as the oil droplet moves from position A to G. Given the designed geometrical parameters, the capacitance variation (C_x_–C_0_) responded to a 1.6 mm long oil droplet is around 1.0 pF for each connection, which is definitely detectable by this sensor. This is the simplest case with only one droplet considered; if more than one droplet appears in the effective sensing area, the capacitance variation will increase. Besides, the trend of capacitance variation for different connections is quite distinct: for one connection, the droplet at positions B and C can affect the capacitance variation; for two connections, the droplet at positions B to E takes effect; and for three connections, the droplet influences the capacitance from position B to F. This indicates that for practical applications, the effective sensing length of the sensor had a better match with droplet length so that a good sensitivity can be guaranteed.

### 4.3. Optical Observation of Droplet Flow

**Figure 7.**
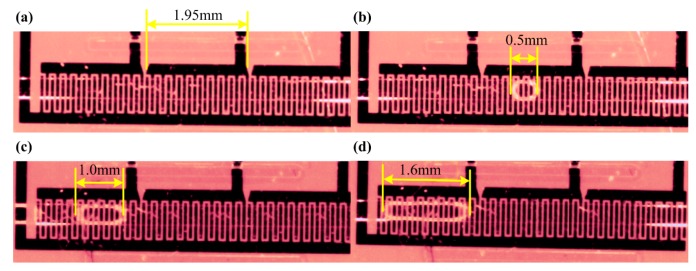
Optical images for oil droplets in continuous water. (**a**) Sensing unit; each gives an effective sensing length of 1.95 mm while connected; (**b**) oil droplet with 0.5 mm length; (**c**) oil droplet with 1.0 mm length; (**d**) oil droplet with 1.6 mm length.

To verify the working principle of the droplet sensor, droplets with different sizes were driven through the sensor and the output signals were recorded. Three different connections (Figure 6a) activated effective sensing lengths of 1.95, 3.95 and 5.95 mm (Figure 7a). Three observed oil-in-water droplets with respective sizes are exhibited in Figure 7. The shortest droplet, 0.5 mm (Figure 7b), was obtained with water as continuous phase at flow-rate of 0.005 mL/min while oil as dispersed phase at flow-rate of 0.017 mL/min. The second droplet, 1.0 mm long (Figure 7c), was with water at a flow-rate of 0.03 mL/min and oil at 0.01 mL/min; and the third droplet, 1.6 mm long (Figure 7d), was with water at 0.02 mL/min and oil at 0.01 mL/min.

By precisely controlling the flow-rates of both water and oil, it was possible to maintain an almost stable oil-droplet sequence with nearly uniform intervals between the droplets, with water as the continuous phase flowing through the microchannel. However, it was also observed that droplet shape was not exactly the same, even with the oil/water two-phase flow-rates well controlled. It should be noticed that the droplets inside the microchannels are somewhat like a crushed “pie” restrained by the channel wall. Therefore the behavior of the droplets was inevitably affected by the microchannel wall. On the one hand, this could be partly attributed to the hydrophobic surface conditions of the microchannel, as both PDMS and SU-8 are hydrophobic, which can lead to a slip-flow boundary in the channel. On the other hand, it can be partly ascribed to the thickness of the IDEs. Even though it is much thinner compared to the thickness of SU-8, the thickness ratio of IDEs to SU-8 is still 1/20, which looks like a spatially periodical “artificial roughness” every 100 μm on the bottom wall. That means the bottom wall of the microchannel was not a perfectly smooth surface due to the IDEs embedded beneath. Due to the forgoing two aspects, the droplets inside the microchanel are easier to deform in profile. Such profile deformation of droplets will cause transient fluctuations in capacitance, which is reflected by the small fluctuations in the output signal.

### 4.4. Voltage and Capacitance Variation—Detection of Oil-in-Water Droplets

Detection of oil droplets was achieved with different connections as shown in Figure 6a. The data were acquired for a time interval of 10 s. It was found that the longer the droplet was, the higher the modulus of the output voltage from the acquisition circuit was. The minimum output voltages for the three observed oil-in-water droplets are shown in Table 3; and the corresponding capacitance value was determined according to the linear equations in Figure 5a.

**Figure 8.**
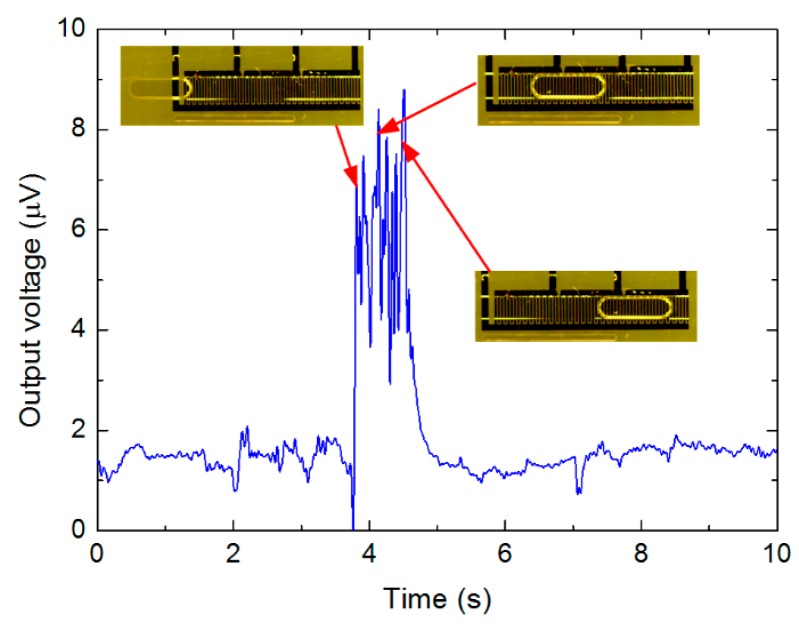
Voltage variation measured with a water droplet 2.1 mm long in continuous oil (flow direction from left to the right), by use of three connections (water flow-rate at 0.007 mL/min and oil flow-rate at 0.003 mL/min).

According to Table 3, the shorter the droplet is, the less the output voltage is, indicating less capacitance variation compared to the initial capacitance. Given the same small droplet for more sensing area (three connections), such capacitance variation is relatively smaller as the initial capacitance for three connections is much larger, therefore the output voltage is much less. This means that for a given droplet passing through the sensor with different sensing area, the induced capacitance variation would be different. In addition to the data listed in Table 3, Figure 8 shows an acquired data sample while a water droplet in continuous oil passing through the sensing area.

According to Figure 8, for the 2.1 mm long droplet with three active connections, the output voltage is approximately around 8.0 μV, and the equivalent capacitance was around 2.95 pF in terms of Figure 5b. In contrast to oil droplet in continuous water, the output voltage increased as the water droplet in oil was detected. When the water droplet entered the sensing area, there was an increase of the output voltage. While the droplet completely stays within sensing area of the sensor, the output voltage maintained at higher levels, with some fluctuations. Finally, when the droplet left the sensing area, the output voltage decreased to initial level.

It can be deduced from Figure 8 that the period for the output voltage variation lasted approximately 1.2 s, which coincided with the time for the droplet to travel through the sensing area. Therefore, it is possible to evaluate the real droplet velocity from the measured data, which might be different from the superficial velocity of each phase calculated from the volume flow-rates set in the syringe pumps. Such conclusion is also valid for the oil droplets entrained in continuous water, or bubbles in continuous liquid flow.

### 4.5. Discussion

The wettability of the channel wall would affect the flow behavior of liquids in microchannels. Inherently, both PDMS (which forms the sidewalls and the top wall of microchannel) and SU-8 [30] insulation film (bottom wall of the microchannel) are hydrophobic, therefore, there would be a slip boundary rather than a non-slip boundary for continuous water flow through the microchannel. However, the influence of the wettability of the hannel wall on the capacitance variation is not very clear so far. Due to the hydrophobic surface, the droplet profile might be affected, consequently influencing the capacitance variation. This could be an interesting topic worthy of further investigation in future work.

The test results on the detection of oil droplets in continuous water or water droplets in continuous oil have demonstrated that the present droplet sensor can work well with two fluids having considerable differences in permittivity. As a capacitive sensor, if there were not so big differences of relative permittivity in the two fluids, the capacitance variation (output voltage) would be rather smaller. Therefore, the geometrical parameters of the interdigital capacitive sensor, e.g., the electrode width, the electrode gap, and the number of electrode pairs would have to be optimized for better performance. That is, the parameters are to be optimized to keep the capacitance variation at the same magnitude as that of the initial capacitance.

It should be noticed that the organic solvents will diffuse into PDMS and cause it to swell [31] if used for a long time period. We observed obvious swelling of PDMS cover after 4 weeks use with olive oil. Therefore, this two-phase capacitive sensor is suitable for gas/water two-phase flow. If organic solvents were involved, this sensor is more preferable as a disposable device for short time usage. Otherwise, other more chemically stable materials have to be found to replace the PDMS cover. For convenience of experimental investigation, typical fluids like the deionized water and olive oil were employed as the test fluids. For specific applications in the biomedical field, some other related fluids shall be tested in the further work.

## 5. Conclusions

In this work, we have demonstrated a capacitive microfluidic two-phase sensor employing IDEs and a thin insulated film. With a simple fabrication processes, the droplet sensor was realized with a multilayered structure. With two immiscible liquids, deionized water and olive oil, the capacitance variation was measured in both simulation and experimental methods. The results verified that the capacitance variation depends on the droplet length and effective sensing length of the sensor. With diverse measurement connections with respect to the droplet length, the sensor can achieve good sensibility. The capacitance variation can be qualitatively determined by the measured output voltage, due to a good linearity between them. Besides, the real velocity of the droplets can also be retrieved from the output voltage. As only simple microfabrication processes were involved, this capacitive droplet sensor is promising for integrateion with lab-on-chip devices, to perform *in situ* detection. This capacitive sensor can also work for bubble detection in liquids as long as the two fluids involved are immiscible with each other and have a distinct related permittivity between them.

## Figures and Tables

**Table 1.** Geometrical parameters of the IDEs in droplet detector.

**Geometrical Features**	**Dimensions**
*a* (μm)	25
*w* (μm)	50
*l* (μm)	850
*n* (per connection)	10

**Table 2.** Initial capacitance of the sensor with different fluids and different active finger pairs.

**Number of Active Finger Pairs**	**Initial Capacitance, *C*_0_(pF)**	

	**Oliver Oil**	**Water**
10	0.73	2.15
20	1.40	4.18
30	2.08	6.11

**Table 3.** Output voltage obtained for each droplets passing the sensor: 0.5 mm length, 1.0 mm length and 1.6 mm length; for each of the droplets, the capacitance value is determined in terms of the fitting equations in Figure 5a.

**Droplet Length**	**1 Connection**		**2 Connections**		**3 Connections**	

	**Output Voltage (μV)**	***C****_x_***(pF)**	**Output Voltage (μV)**	***C****_x_***(pF)**	**Output Voltage (μV)**	***C****_x_***(pF)**
0.5 mm	−1.05	2.03	−0.90	4.06	−0.75	6.01
1.0 mm	−3.92	1.69	−2.66	3.86	−2.48	5.81
1.6 mm	−5.22	1.53	−4.57	3.63	−3.79	5.65

## Nomenclature

***Variables***	**Meaning**	**Unit**	***Greek***	**Meaning**	**Unit**

*a*	Half gap distance	[m]	ε	Permittivity	[F/m]
*C*	Capacitance	[F]	*Subscripts*		
*h*	Height	[m]	*0*	Reference	-
*I*	Current	[A]	*eff*	Effective	-
*l*	Length of the	[m]	*o*	Olive oil	-
*n*	Number of finger	-	*w*	Water	-
*Q*	Volumetric flow rate	[mL/min]			
*R*	Resistor	[Ω]			
*T*	Penetration depth	[m]			
*V*	Voltage	[V]			
*w*	Finger width	[m]			
